# A Bacterially-Expressed Recombinant Envelope Protein from Usutu Virus Induces Neutralizing Antibodies in Rabbits

**DOI:** 10.3390/vaccines9020157

**Published:** 2021-02-16

**Authors:** Kinga Böszörményi, Janet Hirsch, Gwendoline Kiemenyi Kayere, Zahra Fagrouch, Nicole Heijmans, Roberto Rodriguez Garcia, Soesjiel Dwarka, Amy van Dijke, Boyd Aaldijk, Ronald Limpens, Montserrat Barcena, Bram Koster, Babs Verstrepen, Willy Bogers, Clemens Kocken, Gesine Cornellissen, Ernst Verschoor, Bart Faber

**Affiliations:** 1 Department of Virology, Biomedical Primate Research Centre, Lange Kleiweg 161, 2288 GJ Rijswijk, The Netherlands; kayere@bprc.nl (G.K.K.); Fagrouch@bprc.nl (Z.F.); verstrepen@bprc.nl (B.V.); bogers@bprc.nl (W.B.); Verschoor@bprc.nl (E.V.); 2 Department of Biotechnology, Hamburg University of Applied Sciences, Ulmenliet 20, 21033 Hamburg, Germany; janet.medina.hirsch@gmail.com (J.H.); gesine.cornelissen@haw-hamburg.de (G.C.); 3 Department of Parasitology, Biomedical Primate Research Centre, Lange Kleiweg 161, 2288 GJ Rijswijk, The Netherlands; n.heijmans@bprc.nl (N.H.); Rodriguez@bprc.nl (R.R.G.); soesjieldwarka@gmail.com (S.D.); amyvdijke@gmail.com (A.v.D.); boydaaldijk@gmail.com (B.A.); Kocken@bprc.nl (C.K.); 4 Section Electron Microscopy, Leiden University Medical Center, Einthovenweg 20, 2300 RC Leiden, The Netherlands; r.w.a.l.limpens@lumc.nl (R.L.); M.Barcena@lumc.nl (M.B.); A.J.Koster@lumc.nl (B.K.)

**Keywords:** Usutu virus, USUV, vaccine development, flavivirus, emerging disease, recombinant E protein, neutralizing antibodies

## Abstract

Background: Recently, an emerging flavivirus, Usutu virus (USUV), has caused an epidemic among birds in Europe, resulting in a massive die-off in Eurasian blackbirds. Currently found only in Europe and Africa, it can be envisioned that Usutu virus will follow the path of other flaviviruses, like West Nile virus and Zika virus, and will spread via its mosquito vectors and bird hosts to other parts of the world. Several cases of human infections by Usutu virus have already been published. Anticipating this spread, development of an efficacious vaccine would be highly desirable. Method: This study describes the production in *E. coli*, purification, and refolding of a partial USUV envelope protein. Prior to immunization, the protein was characterized using size exclusion chromatography, transmission electron microscopy and dynamic light scattering, showing the limited presence of virus-like structures, indicating that the protein solution is probably a mixture of mono and multimeric envelope proteins. Results: Immunizations of two rabbits with the refolded E-protein fraction, mixed with a strong adjuvant, resulted in the generation of neutralizing antibodies, as evidenced in an in vitro assay. Discussion: The way forward towards a subunit vaccine against Usutu virus infection is discussed.

## 1. Introduction

Usutu virus (USUV) is a positive-stranded RNA virus belonging to the family of *Flaviviridae,* genus Flavivirus [1]. Since its first isolation in South Africa in 1959 from a *Culex neavei* mosquito, a widespread occurrence of USUV has been observed in several countries [2]. The first emergence of USUV in Europe was thought to be in 2001 in Austria, the virus being responsible for a high mortality among Eurasian blackbirds (*Turdus merula*) [3]. However, in a retrospective study, Weissenböck et al. already confirmed the presence of the virus in Italy in 1996, where it caused a high number of deaths in wild birds [4]. Since then, USUV has spread to a large number of European countries, including Hungary [5], Switzerland [6], Czech Republic [7], Spain [8], Germany [9,10], Belgium [11], Poland [12], France [13] and the Netherlands [14].

USUV has an enzootic lifecycle with birds as amplifying hosts and ornithophilic mosquitos as vectors, similar to the epidemiology of the related flavivirus, West Nile Virus (WNV) [6,15]. Humans are considered as incidental, dead-end hosts. Until now, USUV has been described in eight different mosquitos and at least 62 different avian species [16]. The common house mosquito (*Culex pipiens*) is considered to be the main vector in Europe, and among birds, the Eurasian blackbird has shown the highest mortality [5,17,18,19]. The virus may have been introduced into Europe from Africa via (one of) the three main routes of migratory birds through the Iberian peninsula, Italy, and Eastern Mediterranean countries [20]. Interestingly, while it can cause high mortality in various bird species in Europe, it has not yet been associated with bird deaths in Africa [15,21].

Beside avian species, USUV can also cause infections in humans. Infection may be asymptomatic or can be associated with a wide range of symptoms, varying from mild to severe [22,23,24,25,26,27,28]. In total, 22 human clinical cases have been documented until today [26,27]. The first human case of USUV infection was reported in the Central African Republic in 1981, the second in Burkina Faso in 2004. Both patients only developed mild symptoms, including jaundice, fever, and rash [29]. In 2009, the first European cases were reported in two immunocompromised patients suffering from meningoencephalitis [25,30]. Since then, several human disease cases have been confirmed, and serological surveillance studies of blood and organ donors reported the presence of USUV-specific antibodies in serum samples [25,28,31,32,33,34]. 

The recent outbreak of Zika virus in Brazil in 2015, and the spread of West Nile virus across the USA following its introduction to New York in 1999, indicate that the conditions for a rapid spread of flaviviral infections exist in large parts of the world, but also show that such outbreaks are unpredictable [35].

While USUV is not considered to be a major threat to human health at this point in time, it can be anticipated that USUV will further spread over a large part of the world. When this is complemented by an increase in virulence, like that found in West Nile virus, and Chikungunya virus, the health of many people may be at risk [36,37]. For that reason, the World Health Organization (WHO) has put USUV on the list of priority diseases for which no, or insufficient, treatments exist [38]. 

Thus, there is a clear need to be prepared for an USUV outbreak. The development of specific antivirals may be considered [39], but the most cost-efficient method is to prevent such an outbreak by the development of a vaccine. It has been demonstrated that yeast-produced dengue virus envelope (E) protein domains I to III are able to elicit neutralizing antibodies in mice [40]. Here, we describe the production of the USUV E protein in *E. coli*, its purification, and the characterization of the expression product. Subsequently, we assessed the potential of the protein to induce neutralizing antibodies in rabbits.

## 2. Material and Methods

### 2.1. Protein Production

The USUV strain 1477 was used [GenBank acc. no. KJ438705] to amplify the gene of interest. Reverse-transcription was applied on viral RNA that was isolated from virus-infected Vero cell supernatant. The resulting cDNA was used as a template for amplification by PCR, using 5’-CTGCTCATATGTTCAACTGCCTTGGT-3’ as a forward primer, and 5’-TCCAATGTCGACTCCTGCTTTGTGCCA-3’ as the reverse primer. The amplicon sequence codes for amino acids 294 to 693 of the USUV polypeptide, equal to amino acid 1 to 400 of the viral envelope protein, comprising domain I to III.

The amplified fragment was cloned using the *NdeI* and *SalI* restriction sites into the pJExpress412 expression vector (DNA2.0), in the frame with the vector-encoded C-terminal hexa-histidine tag, and transformed to *E. coli* DE3 electrocompetent cells (ThermoFisher, Landsmeer, The Netherlands).

### 2.2. Cultivation of E. coli

Four 500 mL LB broth-containing two-liter flasks were inoculated with 3 mL of overnight culture of the selected clone. When the cultures reached an OD_600nm_ of 0.8 after approximately three hours of incubation at 200 rpm and 37 °C, they were induced with IPTG at a final concentration of 0.5 mM. Three hours later, cells were harvested by centrifugation in a JA14 rotor, at 5000 rpm, for 20 min at 4 °C (Beckman-Coulter, Woerden, The Netherlands). Subsequently, cells were washed once with PBS, pH 7.4, and frozen at −20 °C.

#### Isolation of Inclusion Bodies

Cell pellets were thawed, resuspended in 8 mL lysis buffer (50 mM Tris, pH 8.0, 25% sucrose, 1 mM EDTA), and homogenized with the aid of a Dounce homogenizer. Two milliliters of lysis buffer, containing 10 mg lysozyme and a tablet of EDTA-free protease inhibitor cocktail (cOmplete mini, Roche, Almere, The Netherlands) were added, mixed thoroughly and incubated on ice for 30 minutes. Then, 100 µL 1.0 M MgCl_2_, 20 µL RNase (50 mg/mL; 100,000 units/mg) (Sigma-Aldrich, Zwijndrecht, The Netherlands), 20 µL DNase (50 mg/mL; 400,000 units/mg) (Sigma-Aldrich) and 200 µl 10% NP40 were added. After mixing, the suspension was incubated at 4 °C until the viscous consistency of the suspension was resolved.

Subsequently, 20 mL of detergent buffer (20 mM Tris pH 7.5, 200 mM NaCl, 2 mM EDTA, 1% sodium deoxycholate, 1% NP40) was added, as well as 200 µL of 1.0 M DTT. The suspension was mixed and homogenized using a Dounce homogenizer. The homogenate was centrifuged for 20 min at 4 °C in a Beckman JA14 rotor at 8000 rpm and the supernatant was removed. The pellet was resuspended in 20 mL 10 mM Tris, pH 8.0, 0.5% Triton X-100 (Buffer A), mixed well and homogenized, followed by incubation for 15 min on ice. After centrifugation, the pellet was homogenized/washed again, now using 20 mL of Buffer B, 10 mM Tris, pH 8.0, 0.5% Triton X-100 and 1.5 M KCl and incubated for 10 min on ice. After another washing step, in 20 mL Buffer A, the pellet containing the inclusion bodies was finally washed with 20 mL PBS (10’ incubation on ice) and resuspended in 16 mL Tris-Urea buffer (20 mM Tris, 8.0 M urea, pH 8.0).

### 2.3. Protein Purification

The inclusion body (IB) suspension in urea was thoroughly mixed and homogenized. Subsequently, the solution was centrifuged for 1 hour in a Beckman Ultracentrifuge at 40,000 rpm (Ti70 fixed angle rotor) at ambient temperature. The insoluble fraction (pellet) was discarded.

The supernatant was diluted 1:1 with the Tris-Urea buffer and applied on a 5 mL IMAC FF pre-fab column (ThermoFisher, Landsmeer, The Netherlands). Prior to use, the column was charged with 1.0 mL 0.3 M nickel sulfate, equilibrated with the Tris-Urea buffer containing 10 mM imidazole, and the USUV E protein was eluted from the column (2 mL/min) using 20 mM Tris, pH 8.0, 100 mM imidazole and 8.0 M urea. The fraction containing the purified E protein was concentrated using ultrafiltration filters with a cut-off of 30 kDa (Merck-Millipore, Amsterdam, The Netherlands). The elution buffer was exchanged to 20 mM Tris, pH 8.0, 4.0 M urea. Protein determination was done by µBCA, using BSA as the standard (Sigma-Aldrich, Zwijndrecht, The Netherlands). Protein concentration was adjusted to 2.0 mg/mL using the same buffer.

Densitometry (Bio-Rad Chemidoc, using the manufacturer’s software) was used to estimate the purity of the E protein. This was done after protein separation using SDS-PAGE with pre-cast 4%–12% gradient gel cassettes (Invitrogen, Carlsbad, Germany), using MES buffer as the eluent, followed by staining with Safestain (Invitrogen, Carlsbad, Germany).

### 2.4. Protein Re-Folding

5 mL of the purified USUV E protein solution (2.0 mg/mL in 20 mM Tris, pH 8.0, 4.0 M urea) was added drop-wise to 100 mL of refolding buffer (20 mM Tris-HCl, 50 mM NaCl, pH 8.5), which was vigorously stirred with the aid of a magnetic stirrer. After addition, the solution was (slowly) stirred for another three hours at 4 °C. Next, the buffer solution was replaced by fresh refolding buffer by repeated (5x) concentration/dilution steps, and ultimately concentrated to a volume of 5 mL, using filtration units (Sartorius, Göttingen, Germany) with a cut-off of 30 kDa.

### 2.5. Size Exclusion Chromatography

Analytical Gel filtration was performed on a Superose 12 column (ThermoFisher, Landsmeer, The Netherlands) using an Agilent 1100 series HPLC system with UV detection at 280 nm (Agilent, Amstelveen, The Netherlands). The eluent was identical to the buffer that was used to refold the E protein (20 mM Tris-HCl, 50 mM NaCl, pH 8.5), and the flow-rate was 0.5 mL/min.

### 2.6. Transmission Electron Microscopy

Purified E protein samples that were eluted in the void volume of the size exclusion chromatography were examined by electron microscopy. First, the samples were mildly fixed for 30 min with 1.6% (*w*/*v*) paraformaldehyde and immediately applied to freshly glow-discharged copper grids with a carbon-coated pioloform layer. After 1 min, the samples were blotted and negatively stained with a 2% phosphotungstic acid (PTA) solution for 30 s. After blotting away the excess PTA solution and drying, the samples were examined in a Tecnai 12 Twin electron microscope (Thermo Fisher Scientific (formerly FEI), Waltham, MA, USA) equipped with a OneView 4k high-frame-rate CMOS camera (Gatan, Pleasanton, CA, USA), and operated at 120 kV.

### 2.7. Dynamic Light Scattering

300 µL of the sample was analyzed in polystyrene cuvettes with a Zetasizer Nano ZS (Malvern Instruments, Malvern, UK) at a wavelength of 632.8 nm and a detection angle of 173°. After a 2 min equilibration time at 22 °C, three measurements with 15 runs of 10 s each were performed. The shown particle distribution profile was intensity weighed.

### 2.8. Immunization of Rabbits

Two rabbits were immunized four times with 1.0 mL (100 µg/mL) refolded USUV E protein, using the speedy immunization protocol with immunizations at days 0, 7, 10, and 18 with a final bleeding at day 28. The adjuvant(s) used were proprietary and their compositions were not disclosed. The animal work was carried out at Eurogentec S.A. in Seraing, Belgium, in accordance with the 2010/63/EU directive on the protection of animals used for scientific purposes. The protocols were approved under reference CE/SANTE/E/001 by the CER ethical licensing committee.

### 2.9. Isolation of Total IgG

Total IgG from 10 mL of serum from each rabbit was purified using Protein G agarose (GE Healthcare, Eindhoven, The Netherlands) according to the manufacturer’s instructions.

### 2.10. Enzyme-Linked Immunosorbent Assay

An ELISA was performed using rabbit serum samples in 96 well flat-bottomed microtiter plates (Greiner, Alphen a/d Rijn, The Netherlands), coated with 1 µg/mL purified USUV E-antigen, according to published methods [41]. Alternatively, inactivated concentrated culture supernatants from virus infected Vero cells were used to coat the plates. The secondary antibody for total IgG was goat anti-rabbit IgG conjugated to alkaline phosphatase (Pierce, Rockford, US). Titers are expressed as arbitrary units, where 1 AU yields an OD of 1 over the background (serum against non-related protein). Thus, the amount of AU of a sample is the reciprocal dilution at which an OD of 1 over the background will be achieved.

### 2.11. Dot Blot

Vero cell supernatants containing USUV particles were concentrated as 10-fold and subsequently 3-fold dilution series and were made in PBS. From the dilutions, 3 µL was applied on a nitrocellulose membrane and left to dry. The membrane was submerged in blocking buffer (as described above) and developed in a similar fashion as a western blot, using a 1:100 dilution of the rabbit serum as a primary antibody, and AP-conjugated goat anti-rabbit antibody as the secondary antibody 1:3000 (Pierce, Rockford, IL, USA). A mixture of NBT (0.33 mg/mL) and BCIP (0.17 mg/mL) (Promega, Leiden, The Netherlands) in 100 mM Tris-HCl, 150 mM NaCl, and 1 mM MgCl_2_ buffer pH 9.0 was used to detect the AP conjugate.

### 2.12. Virus Stocks

Two different USUV strains were used for the experiments, lineage Europe 3 strain 1477 (GenBank acc. no. KJ438705) and Africa 3 isolate S.nebulosa-6004/NL/2016 (GenBank acc. no. KY128482). Besides, WNV lineage 1 (GenBank acc. no. GU011992) and 2 (GenBank acc. no. KF179640), DENV serotype 2 (GenBank acc. no. KU725663) and ZIKV (GenBank acc. no. DQ859059) were used for the microneutralization assays. Both USUV strains and both WNV lineages were propagated on Vero and C6/36 cell lines, DENV and ZIKV on the Vero cell line. Gibco Minimum Essential Media (MEM) supplemented with 10% fetal bovine serum (FBS), penicillin (100 U/mL), and streptomycin (100 μg/mL) was used as culture medium. All virus stocks were titrated in 100 µL M10 and inoculated on Vero cells in triplicates to determine the TCID50 with the Reed and Muench method [42].

### 2.13. Microneutralization Assay 

Serum isolated from USUV E protein-immunized rabbits was diluted 1:16 in culture medium and 100 µL was transferred into the first row of a U-shaped 96-well plate (Greiner, Alphen a/d Rijn, The Netherlands). A two-fold serial dilution was performed in 100 µL culture medium in triplicates (dilutions 1:10 to 1:2048), and 100 µL of virus containing 100 TCID_50_ was added to each well. After 2 hours of incubation at 37 °C, the serum-virus mixture was transferred to a monolayer of Vero cells in a flat bottom 96-well plate and incubated again for 2 h at 37 °C. Subsequently, the supernatants were removed and replaced with fresh culture medium. The plate was then incubated at 37 °C. Each plate included both a virus control (no serum) and a cell control (no virus, no serum). The assay was carried out in triplicates. Viral infection of the Vero cells was then determined using an indirect ELISA. At six days after infection, the plates were washed with PBS and fixed with cold, 80% acetone. Then, plates were air-dried and viral proteins were detected with an indirect ELISA using anti-envelope mAb (Anti-Flavivirus Group Antigen Antibody, clone D1-4G2-4-15) (1:2000, Merck KGaA, Darmstadt, Germany) containing filtered BSA (bovine serum albumin) to block unspecific binding and goat anti-mouse HRP antibody (1:2000, KPL, Gaithesburg, MD, USA). The reaction was developed with OPD (o-phenylenediamine dihydrochloride) (Sigma-Aldrich, Zwijndrecht, The Netherlands) and stopped with sulfuric acid. Absorbance was measured at 490 nm [43]. The neutralizing titers were calculated using the adapted method by Reed and Muench [42].

## 3. Results

### 3.1. Protein Production and Purification

**Figure 1:**
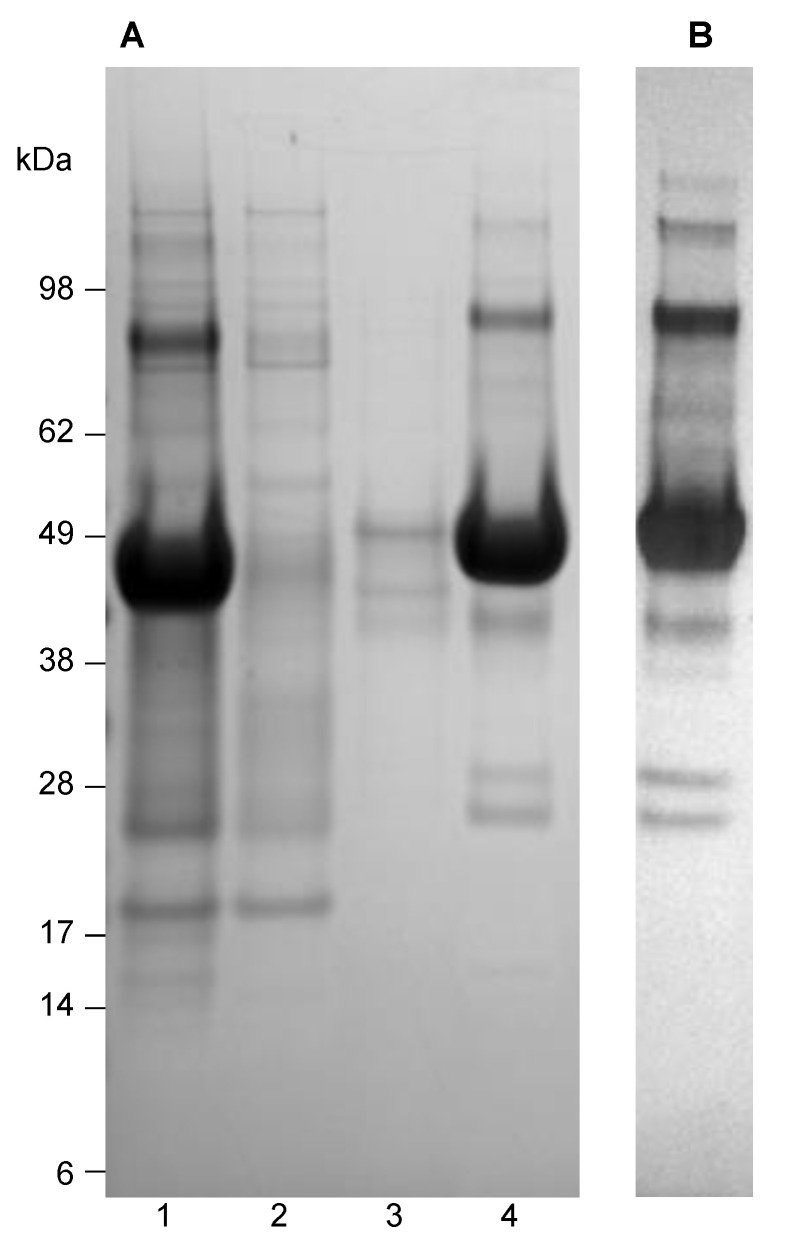
(**A**) Coomassie-stained SDS-PAGE gel showing the purification procedure of USUV E protein from *E. coli*, starting with isolated inclusion bodies. Lane 1, inclusion bodies solubilized in 20 mM Tris, 8.0 M urea, pH 8.0, prior to application on the Ni-IMAC column; lane 2, Flow-through; lane 3, wash with 20 mM Tris, 8.0 M urea pH 8.0, 10 mM imidazole; lane 4, elution with 20 mM Tris, 8.0 M urea pH 8.0, 100 mM imidazole. (**B**) Western blot analysis of the purified USUV E protein (as in lane 4 of panel A), developed using mouse anti-HIS as the primary antibody.

USUV E protein domain I to III (aa. 1 to 400) was successfully expressed in *E. coli*. In batch culture, an expression level of 70 mg/L was reached without optimization. Using densitometry of the SDS-PAGE gel (data not shown), it was estimated that 40% of the total bacterial protein was the USUV E expression product (Table 1), while SDS-PAGE also showed that the expressed protein was close to its expected theoretical molecular mass of 44 kDa (Figure 1).

A significant amount of E protein was detected in the inclusion body (IB) fraction. More than half (60%) of the E protein was lost during the isolation of the IB, combined with a limited gain in purity over the whole cell extract. Solubilization in 8.0 M urea, followed by ultracentrifugation, again resulted in a significant loss in yield. However, this step cannot be avoided because column chromatography is greatly complicated without clarification of the solutions.

Nickel-activated Immobilized Metal Affinity Chromatography (IMAC) of the subsequent fraction resulted in a significant increase in purity without a significant loss in the protein (Table 1, Figure 1).

Ultimately, a yield of 16 mg recombinant E protein with a purity between 80–90% was obtained, which was 6% of the estimated total starting amount of expressed E protein. Western blot analysis of the purified fraction revealed that the remaining impurities were probably product-derived as these products bound to the anti-His-tag antibodies. If the product-derived impurities were excluded, the purity level would go up to over 90% (Figure 1).

Next, a simple re-folding procedure was applied on the purified E protein fraction, at a fixed protein and urea concentration. Re-folding was done by rapid dilution using a 1:20 dilution ratio. Buffer replacement to the immunization-compliant refolding buffer was achieved by repeated concentration/dilution steps using diafiltration units. 

### 3.2. Protein Analysis

For other flaviviral vaccines, it has been shown that the conformational integrity of the E protein is crucial for the induction of neutralizing antibodies [44,45]. Therefore, the conformational state of the E protein in solution was investigated further, bearing in mind that the flaviviral E proteins may spontaneously assemble into VLPs, as observed by Mani et al. [40].

**Figure 2:**
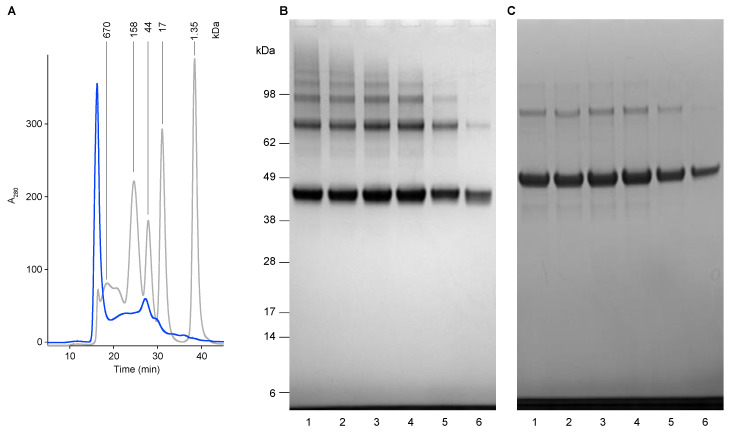
(**A**) Size-exclusion chromatography of USUV E protein fraction after a refolding procedure using rapid dilution (blue line), compared to a mixture of protein markers (grey line) with their molecular mass indicated. (**B**) SDS-PAGE analysis (non-reduced) of collected 1 mL fractions of the SE-chromatography. Lanes 1; fraction 16–18 min; lanes 2, 18–20 min; lanes 3, 20–22 min, lanes 4, 22–24 min, lanes 5, 24–26 min, and lanes 6, 26–28 min. (**C**) SDS-PAGE analysis (non-reduced) of collected 1 mL fractions of the SE-chromatography. Lanes are identical as described for Panel B.

The refolded protein fraction was analyzed using Size-Exclusion chromatography (SEC) (Figure 2). The chromatogram shows a significant peak at the void volume (8 mL/16 min) of the analytical SE-column, suggesting that particles may be present. However, analysis of the fractions obtained by SEC by SDS-PAGE analysis showed that all fractions contain the USUV E protein expression product only (Figure 2B,C), while the fractions eluted at smaller volumes (higher Mw) contain a higher degree of multimeric forms (Figure 2B), which disappear after the addition of the reducing agent DTT (Figure 2C), suggesting that disulfide bridges may be involved in the multimerization of the E proteins. The observed pattern suggests that the E proteins interact with each other and form di- and multimers with variable size, eventually to sizes larger than the exclusion limit of the SE-column. 

**Figure 3:**
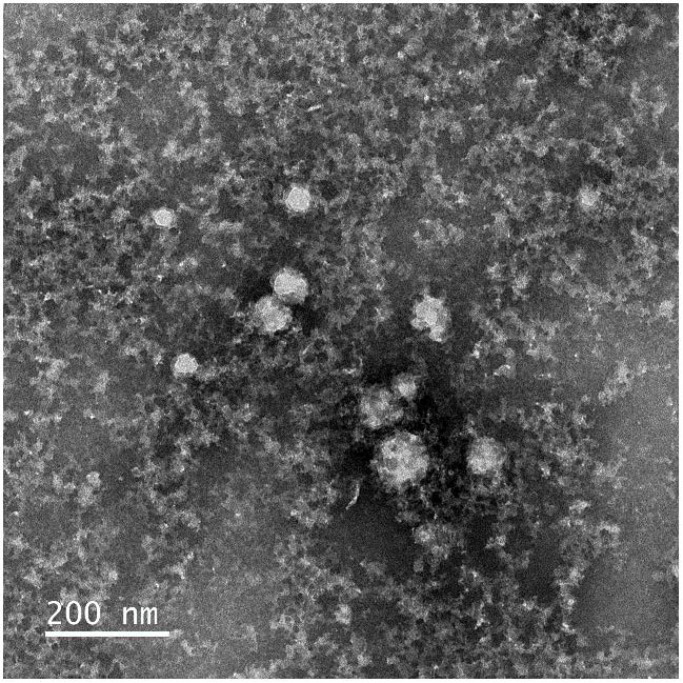
Electron microscopic analysis of purified USUV E protein. Purified USUV E antigen was negatively-stained with PTA and examined under EM. The scale bar indicates a size of 200 nm.

The presence of larger structures was confirmed by transmission electron microscopy of negatively stained samples (Figure 3), but these were only visible after mild fixation with paraformaldehyde. The average size of these particles was estimated to be 47 nm (n = 59, SD = 14 nm), but varied extensively between 20 and 88 nm. Moreover, these particles did not show a highly-ordered structure and were present in low numbers. They were surrounded by material with a milky appearance.

**Figure 4:**
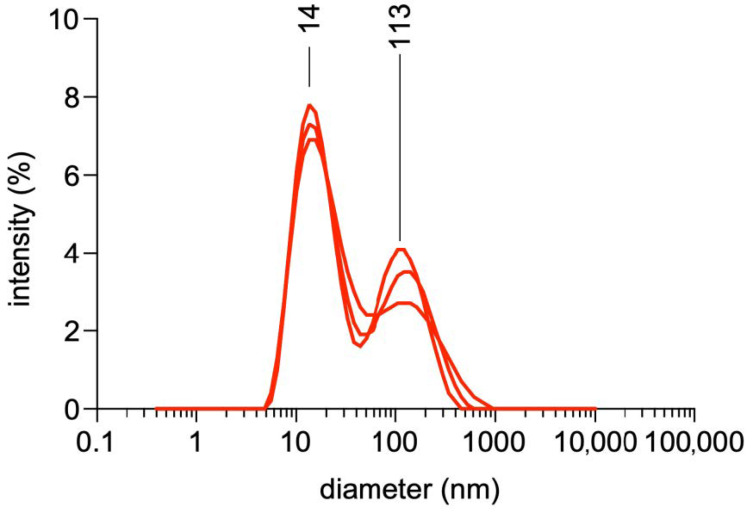
Dynamic Light Scattering analysis of the USUV E protein fraction showing the intensity-weighed particle distribution profile.

DLS analysis of the E protein solution (Figure 4) showed two maxima at 14 and 113 nm, respectively, confirming the lack of uniformity of the particles present, if any.

### 3.3. Analysis of the Humoral Immune Response in Rabbits

**Figure 5:**
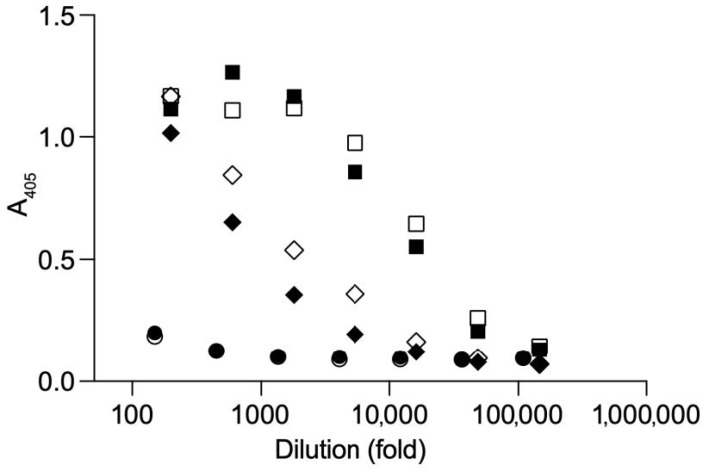
ELISA of two rabbit sera obtained after immunization, including 2 control sera after immunization with a control protein purified using a highly similar procedure. Squares represent day 28 (final bleed) sera from USUV E protein immunized animals, open symbol is serum 710, closed is serum 711. Diamonds represents 2 sera of control protein immunized animals, for which the protein was purified in a highly similar fashion. Circles represent pre-bleed sera from rabbits 710 (open) and 711 (closed). Starting dilution was 1:150.

Irrespective of the lack of highly-ordered structures, let alone VLPs, the refolded E protein fraction was mixed with an adjuvant and used to immunize two rabbits. The sera of the final bleeds (obtained after 28 days) were analyzed in several ways. Firstly, an ELISA was performed to confirm that the produced proteins were recognized by the polyclonal antibodies directed to the bacterial expression product. The ELISA titer, defined as the dilution at which an OD_405_ of more than twice the background was obtained, was determined to be 1:150,000 (Figure 5). Of note, a significant antibody response was observed against the bacterial background.

**Figure 6:**
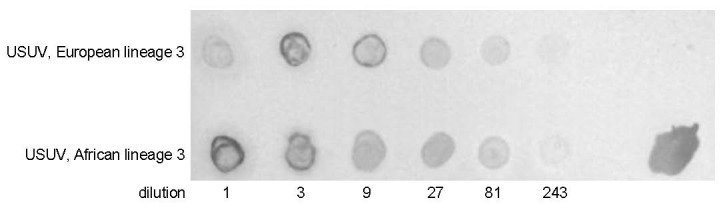
Dot blot of heat-inactivated supernatants of Vero cell-grown USUV strains, stained with pooled rabbit serum of USUV E protein-immunized rabbits. Upper row: USUV, European lineage 3 isolate; lower row, supernatants of USUV, African lineage 3. Supernatants were concentrated and spotted in a 1:3 dilution series. The spot to the right is a positive control, containing the immunogen (*E.coli*-produced USUV E protein).

Secondly, a dot-blot was done to show that native USUV was recognized by the antibodies in the sera. Virus-infected, heat-inactivated Vero cell supernatants were used for this purpose. Two USUV strains were used in this assay, one belonging to the European lineage 3, the other to the African lineage 3. Both showed similar responses on the dot blot (Figure 6).

Thirdly, the two rabbit sera (samples 710, 711) were tested for their functionality in a microneutralization assay. The sera showed neutralizing capacity against the European strain propagated on Vero and C6/36 cells and against the African strain propagated on Vero and C6/36 cells, up to a dilution of 1:128, 1:64, 1:64 and 1:32, respectively. The sera were also tested for neutralization against other Flaviviruses, namely West Nile virus (WNV) lineages 1 and 2, Zika virus (ZIKV) and Dengue virus serotype 2 (DENV-2). Interestingly, high neutralizing capacity against WNV-1 and -2 (up to dilution 1:128 and 1:32, respectively) was found, but no neutralizing activity against the more distantly-related flaviviruses, ZIKV and DENV (Table 2).

## 4. Discussion

Currently, USUV is not considered to be an immediate threat to human health. However, because of its clear potential to generate a public health emergency, and the lack of prophylactic vaccine and therapeutics, the World Health Organization has put this virus on the list of 20 priority pathogens [38]. Indeed, the situation, nowadays with USUV infections in humans, is reminiscent of that of the genetically closely related West Nile virus in the 1990s—few human cases and the virus primarily causing disease in birds. Gradually, more is known regarding the number of subclinical human USUV infections and the steady spread of the virus and its mosquito hosts, especially in Europe. A considerable number of infections in Europe first attributed to West Nile virus are, in retrospect, caused by USUV, while the intensified screening of human sera also resulted in the detection of numerous new human asymptomatic USUV infections [28,32,33,34,46].

The fact that USUV indeed follows the same route that WNV and ZIKV have taken in recent history, the preparative development of a vaccine against USUV infection would be highly opportune. In the present study, we show that USUV E protein is expressed in *E. coli* to high levels, and that the immunization of rabbits with the purified and refolded E protein results in the elicitation of strong humoral immune responses (estimated titer of 1:150,000), including neutralizing antibodies.

The expression level of the USUV E protein in *E. coli* was 70 mg per liter in batch culture. This is a good starting point for further optimization procedures and the production in large-scale fermenters in order to obtain the protein expression levels needed to be able to start GMP production of an USUV vaccine at reasonable costs.

There is room for improvement in the purification procedure as the final recovery is low. The purity of the protein (~80–90%) that was obtained approximates the level needed for clinical testing (>95%). Assuming that most of the impurities are product-derived (based on the response with anti-His-Tag antibodies), the purity level might be significantly increased by prevention of proteolytic processing during purification and refolding procedures. Additionally, the use of an additional chromatographic step, like size-exclusion chromatography, may improve the purity to a level greater than 95%.

Analytics performed on the protein product show that larger structures are formed after the rapid-dilution refolding procedure. These larger structures are not likely to be particles, let alone VLPs. After mild fixation, TEM showed the presence of a limited number of sphere-like particles, close to ~50 nm in size, but not only these varied extensively in diameter (20–88 nm). The particles were amidst a much larger proportion of amorphous material, likely to represent protein aggregates. Calculation of the number of spots expected in the sample (assuming 180 proteins per VLP), based on the protein concentration of the E protein that was used, showed that the large majority of the proteins was not included in these particles.

SEC, followed by SDS-PAGE, showed that the purified refolded E protein fraction consists of the E protein only, partly monomeric, but the majority in a variable state of di- or multimerization and perhaps even aggregation, in which disulfide bridge formation may play a role.

The DLS analysis shows that two peaks with maxima at 14 and 113 nm are present in the protein solution, again suggesting a non-uniform distribution. Both maxima are far from the average particle size observed by electron microscopy, further complicating the interpretation of the data. DLS data showing more than one peak are generally difficult to interpret, as even a very small number of large particles may prevent smaller particles from being detected or at least decrease the intensity they provoke, resulting in an alleged shift in the average diameters.

We conclude, on basis of the TEM and DLS data, that the USUV E protein fraction that was obtained after SEC and used for immunization, consists in part of the monomeric E protein, but mainly in poorly defined E-protein multimers with some ill-defined particles. 

Despite this, the USUV E protein fraction is able to induce USUV neutralizing antibodies in rabbits in the presence of a strong adjuvant. The sera were able to neutralize both European and an African USUV strains, the former at a slightly higher dilution, likely because this strain expresses the homologue protein used to immunize the rabbits. The neutralizing titer against USUV (and WNV, see below) viruses may be regarded as “high”, as in general for viruses, e.g., JE, influenza and smallpox virus, serum dilutions in the range of 1:10 to 1:40 are considered to have protective capacity [47]. However, these are never rock-solid correlations as individuals with titers in this range are not always protected and individuals with lower titers may be protected, most likely due to contributions of innate and adaptive cellular immune responses [48].

Interestingly, the sera were also able to neutralize both WNV lineages, WNV-1 to a similar extent as observed for USUV, but no neutralizing activity was found against ZIKV nor DENV-2. This may be explained by the relatively high similarity of USUV and WNV envelope proteins. The pairwise alignment of the E protein amino acid sequences, USUV strain 1447 shows 78.2% and 77.4% identity with WNV-1 and -2, respectively, and a lower similarity to ZIKV (54.2%) and DENV-2 (47.5%).

Even though USUV and WNV are quite closely related, the strong cross-strain neutralization titer is unexpected. While the comparable neutralizing capacity between USUV and WNV-1 suggests that the epitopes on USUV and WNV recognized by neutralizing antibodies are conserved, the sequence identity of almost 80% still translates to approximately 80 differences with a total length of 400 amino acids. Part of the non-identical amino acids resides in the interior of the protein, another part faces the inside of the virion, and yet another part will be on the interface of two E proteins, forming the dimer and larger structure of the virion, and thus will not be the target of neutralizing antibodies. However, a number of non-identical amino acid residues (identified from the WNV E protein structure [49]) are located on the surface of the E-protein, leading to non-identical epitopes on the WNV protein compared to the USUV E protein. Therefore, a plausible explanation for the high cross-strain neutralization is the existence of dominant “neutralizing” epitopes that are shared between USUV and WNV-1 and WNV-2. Irrespective of this, these findings warrant further research into the possibility for the development of a single vaccine against both WNV and USUV.

Similar to the work described here, we expressed E proteins of related mosquito-borne flaviviruses in *E. coli*. In contrast to the E protein of USUV, the E protein of dengue virus serotype 1 (DENV-1) formed better defined particles, albeit under relatively high pH conditions [50], which were addressed as “virus-sized” particles. The reason why the recombinant DENV-1 E protein formed better-defined particles compared to its USUV counterpart is unclear. Interestingly, immunization of rabbits with these DENV-1 particles, using an identical immunization protocol, did not result in the formation of neutralizing antibodies [50].

The promising results with the recombinant USUV E protein warrant further research into the efficacy of this potential USUV vaccine. A first step could be the testing of the prototype vaccine in interferon alpha/beta receptor-deficient AG129 (IFNAR) mice [51]. If successful, this may be followed by studies in non-human primates (NHP) to test for safety and efficacy, as NHP are hosts for many flaviviruses, presumably also for USUV.

However, before further investigation into the efficacy of the product can take place, the production procedure should be made to be more efficient and the poorly defined state of the final product should be improved. The first step towards this would be the addition of (part of) the membrane protein to promote the assembly of the proteins into VLPs [40]. This has the advantage of promoting the correct conformation of the envelope protein(-dimer), deemed to be essential for flaviviral vaccines in order to induce neutralizing antibodies [52]. Moreover, VLPs may not need a strong adjuvant or one or more booster immunizations, as they have intrinsic immune-stimulatory capacity. Finally, VLPs have advantages over life-attenuated vaccines [53].

## 5. Conclusions

This study shows that the USUV envelope protein expressed in *E. coli* induces neutralizing antibodies against 2 USUV strains in rabbits. Interestingly, neutralizing titers were also observed against the closely related WNV. The results are promising and warrant further research into the protective efficacy of this formulation.

However, before this product may be considered to be a true vaccine candidate, a number of improvements need to be made. For example, the purification procedure should be improved with regards to the yield and the ill-defined character of the protein solution should also be improved. Moreover, the protein should be tested with an adjuvant that is suitable for human use.

## Figures and Tables

**Table 1:** Purification table for USUV E protein.

STEP	Vol. (mL)	Total Protein (mg)	Purity (%) *	Yield (%)	Yield USUV ** E Protein (mg)
Cells	3.4	332	42/(40)	(100)	139
Inclusion bodies (IB)	20	126	51/(46)	38	64.2
Ultracentrifuge Supernatant	19	30	59/(52)	9	17.7
IMAC	40	20	80/(89)	6	16.1

* Purity was estimated from 2 independent purifications. Calculations were made on basis of the first. ** Cultivation volume 2 L batch culture.

**Table 2:** Results of the microneutralization assay. Numbers show the highest dilution of the sera where the infection of the cells was blocked by neutralizing antibodies.

Virus Name	USUV-Eur-3 on Vero	USUV-Eur-3 on C6/36	USUV-Afr-3 on Vero	USUV-Afr-3 on C6/36	WNV-1 on Vero	WNV-1 on C6/36	WNV-2 on Vero	WNV-2 on C6/36	ZIKV	DENV-2
serum 710	1:128	1:64	1:64	1:32	1:128	1:128	1:32	1:32	-	-
serum 711	1:64	1:32	1:64	1:16	1:64	1:32	1:16	1:16	-	-

## Data Availability

The data presented in this study are available in this article.
